# Outcomes of Patients with an Intermediate-Risk Group According to the Japanese Risk Classification of Papillary Thyroid Carcinoma

**DOI:** 10.1007/s00268-023-07073-7

**Published:** 2023-06-02

**Authors:** Kiyomi Horiuchi, Mikiko Fujimoto, Kamio Hidenori, Yusaku Yoshida, Eiichiro Noguchi, Yoko Omi, Takahiro Okamoto

**Affiliations:** 1 Department of Endocrine Surgery, Tokyo Women’s Medical University, 8-1 Kawada-cho Shinjuku-ku, Tokyo, 1628666 Japan; 2 Department of Breast Surgery, Tokyo Women’s Medical University, 8-1 Kawada-cho Shinjuku-ku, Tokyo, 1628666 Japan

## Abstract

**Background:**

The management of intermediate-risk group of papillary thyroid cancer (PTC) is still vague, particularly regarding whether or not total thyroidectomy, postoperative radioactive iodine ablation (RAI-a), and postoperative TSH suppression are mandatory.

**Methods:**

This retrospective study evaluated 680 PTC patients from 2010 to 2017, who were classified into the three risk groups as low, intermediate, and high-risk groups according to the criteria of the Japanese Association of Endocrine Surgeons (JAES) 2010 and underwent surgery according to the JAES guidelines. We retrospectively collected patient data for analyses of disease-free survivals in the intermediate-risk group patients.

**Results:**

We performed surgery on 680 PTC patients from 2010 to 2017. Of them, 297 were classified as the intermediate-risk group. DFS was not statistically significantly different in patients with/without total thyroidectomy and postoperative TSH suppression therapy. For RAI-a, DFS (95% confidence interval) at 3, 5, and 8 years were 93.2% (84.6 ~ 97.2), 81.6% (68,3 ~ 90.2), and 70.7% (51.4 ~ 84.6) in patients with postoperative RAI-a and 100%, 100%, and 100% in patients without postoperative RAI-a after total thyroidectomy, respectively. DFS of patients without RAI-a was superior to those with RAI-a (*P* < 0.0004). Multivariable analysis by stepwise selection method revealed that postoperative RAI-a was a risk factor with a hazard ratio of 5.69. (95% CI 1.998–16.21) (*P* = 0.001131).

**Conclusions:**

Our study did not show the efficacy of RAI-a in patients with intermediate-risk PTC. This study implies that judging the efficacy of adjuvant therapy such as RAI or TSh suppression in intermediate-risk patients is difficult.

## Introduction

Papillary thyroid cancer (PTC) is a slow-growing malignant tumor with an incidence rate of 1.3 per 100,000 in Japan [1]. Although it is evident that surgical resection is needed to treat apparent PTC, the surgical and postsurgical strategy in Japan had been different from that in Western countries. In the 20th century, many Japanese surgeons tried to retain as much of the thyroid gland as possible to maintain thyroid function at a time. In Western countries, total thyroidectomy was a routine procedure at that time [2]. One of the reasons was that postoperative radioactive iodine (RAI) treatments were limited to patients who underwent total thyroidectomy in Japan. The bed for the RAI therapy was lacking because of the small amount of reimbursed hospital fees for RAI therapy [3].

**Fig. 1:**
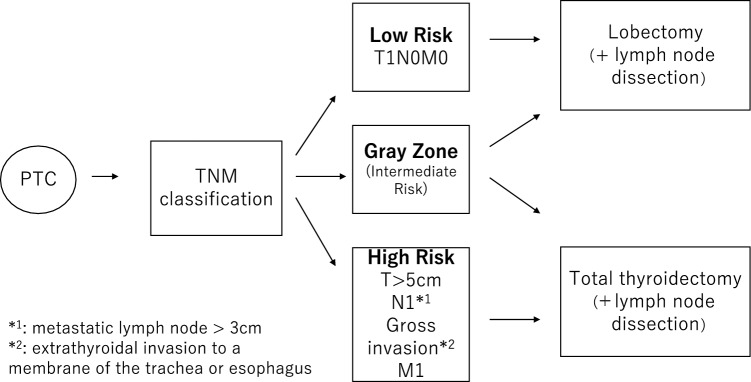
Diagram of Japanese Guidelines for the Management of Thyroid Tumors in 2010

However, the surgical strategy for low-risk PTC has evolved toward the Western, and Japanese surgeons have been getting closer to each other regarding surgical and postsurgical strategies since the ATA guideline was updated in 2015 [4, 5]. Since 2009, outpatient radioiodine ablation has been approved in Japan. Although Kusakabe et al. revealed the safety of 30 mCi radioiodine ablation at an outpatient clinic, the necessity of radioiodine ablation to reduce the risk of recurrence has been questioned by thyroid surgeons [3, 6]. Consequently, the Japanese Association of Endocrine Surgeons (JAES) established a guideline for thyroid tumors and suggested a surgical strategy based on the recurrent risk classification in 2010 [7], which includes low, intermediate-, and high-risk groups. In this guideline, lobectomy is recommended in patients in the low-risk group (Tumor diameter =  <2 cm and negative for lymph node metastasis), and total thyroidectomy in the high-risk group (Tumor diameter >5 cm, or palpable lymph node metastasis larger than 3 cm in diameter, or extra-thyroid extension beyond the membrane to the trachea/esophagus) (Fig. 1). Total thyroidectomy followed by radioactive iodine (RAI) therapy is recommended in the high-risk group, while RAI is not recommended in the low-risk group. The patients classified as neither low nor high-risk groups belong to the intermediate-risk group, and the surgical strategy and postoperative RAI have not been settled. Although the recommendations of the JAES guidelines are based on available evidence and expert consensus, a few studies verified the validity of it. Recently, Ito et al. examined the validity of the revised JAES 2018 guidelines [8, 9]. The strength of their study was a large sample size with a long-term follow-up, while the drawback was that the employed management days before 2004 did not fit the guidelines published in 2018.

We report our retrospective study that adopted the surgical strategy of the risk classification for thyroid surgery in the JAES 2010 guidelines.

This study aimed to analyze the patients’ outcomes of intermediate-risk PTC classified according to JAES guidelines.

## Materials and methods

In this retrospective study, we adopted the management strategies based on the classification of PTC according to the JAES guidelines of 2010. We retrospectively collected the data of all PTC patients who underwent thyroidectomy from 2010 to 2017, after dividing the patients into three risk groups: low, intermediate, and high risk, according to JAES guidelines described above.

The preoperative examination included three examinations, an ultrasound examination (US) of the neck, a fine needle aspiration biopsy to confirm PTC (or lymph node metastasis), and a chest CT scan to detect mediastinal lymph node and lung metastases.

We choose total thyroidectomy for patients classified as high-risk and lobectomy for patients considered as a low-risk group according to the JAES classification. For lymph node dissection, central neck dissection was performed in all the cases (Fig. 1). However, we did not perform lateral neck dissection prophylactically in all patients. A therapeutic lateral neck dissection was performed if fine-needle aspiration of a lymph node revealed thyroid cancer metastasis. Generally, we confirm extra thyroid extension intraoperatively. Suppose the gross extra-thyroid extension is recognized, such as the extension to the membrane of the trachea or esophagus intraoperatively. In that case, total thyroidectomy is performed in a high-risk group. Administration of 30 mCi radioiodine ablation to intermediate-risk patients is at the discretion of the outpatient physician. The outpatient physician follow a Whole-body scan with 10 mCi radiotracer dose to confirm the disappearance of the remnant thyroid gland, Before RAI administration, we gave the patients recombinant human thyrotropin alpha (thTSH: ThyrogenR, Sanofi Genzyme, Cambridge, MA, US) to attain increased TSH levels [10]. Some patients in whom a hot spot remained after RAI-a, as seen by the 10 mCi whole-body scan, received 100 mCi radio-isotope therapy. In Japan, 100 mCi treatment is performed after thyroid hormone withdrawal, and it is prohibited to use thTSH. We defined TSH suppression as suppression of less than half the TSH average range level.

The outcome of this study was disease-free survivals (DFS) estimated using the Kaplan–Meier method and compared by the log-rank test. In the definition of DFS, the day of surgery was considered day 0, and the latest visiting day of any department at our outpatient clinic was considered the last. We defined “recurrence” when the ultrasonography or CT scan detects structural findings. Without structural results, we did not define elevated thyroglobulin (Tg) as a recurrence. We described continuous variables as the median values with range.

Furthermore, we assessed them using the independent-sample *t-*test, while dichotomous variables were evaluated using Fisher’s exact test. For multivariable analysis, we analyzed a Cox proportional hazard model. We used JMP R PRO 16.0.0 (SAS Institute, Cary, NC, USA) for analysis. Statistical significance was set at *p* < 0.05.

The institutional review board of Tokyo Women’s Medical University approved this clinical study (# 4842), and the study was completed following the Declaration of Helsinki as revised in 2013.

## Results

From 2010 to 2017, we extracted data from 688 patients with PTC, among whom eight patients were excluded for the following reasons: three for incidental cancer, two who were not diagnosed as PTC preoperatively, and three with recurrence. Consequently, 680 patients were enrolled in this study. The median follow-up period of all the patients was 37.9 months (range: 2 days ~ 103.4 months). The number of patients, median age, and follow-up period in the low, intermediate-, and high-risk groups were: 276, 297 and 107, 52, 50, and 51 years, and 42.2 (1167), 45.4 (1206), and 37.7 (947) months (days), respectively. In the intermediate-risk group, 128 patients underwent lobectomy, and 169 underwent total thyroidectomy. Ninety-three patients underwent RAI-a in the intermediate-risk group, and only two patients (2.1%) were treated again in 100 mCi. Table 1 shows the patients’ characteristics. Patients were categorized by TNM classification according to Union for International Cancer Control (UICC 7th). In the intermediate-risk group, the recurrence occurred in 18 patients. Of these, 12 patients (66.6%) had lymph node metastasis, and 3 patients (16.6%) had local recurrence, 3 patients (16.6%) had lung metastasis (Table 2).

**Table 1:** Patients’ characteristics

Risk		High	Intermediate	Low
Number		107	297	276
Age: median (range)		51 (19 ~ 89)	50 (15 ~ 87)	52 (21 ~ 84)
Follow up date (days): median (range)		947 (5 ~ 2982)	1206 (5 ~ 3101)	1167 (2 ~ 3063)
pathological *T* (%)	T0 *T1a* *T1b* *T2* *T3* *T4a* *T4b* *TX*	0 (0) 6 (5.6) 21 (19.6) 16 (14.9) 44 (41.1) 18 (16.8) 1 (0.9) 1 (0.9)	0 (0) 44 (14.8) 86 (28.9) 156 (52.5) 7 (2.4) 2 (0.7) 0 (0) 2 (0.7)	1 (0.4) 113 (40.9) 162 (58.7) 0 (0) 0 (0) 0 (0) 0 (0) 0 (0)
pathological *N* (%)	*N0* *N1a* *N1b*	35 (32.7) 2 (1.9) 70 (65.2)	152 (51.2) 31 (10.4) 114 (38.4)	276 (100) 0 (0) 0 (0)
*M* (%)	*M0* *M1*	93 (86.9) 14 (13.1)	297 (100) 0 (0)	276 (100) 0 (0)
Stage (%)	I II III IVA IVB IVC	39 (36.4) 2 (1.9)) 9 (8.4) 42 (39.3) 1 (0.9) 14 (13.1)	141 (47.5) 54 (18.2) 20 (6.7) 82 (27.6) 0 (0) 0 (0)	276 (100) 0 (0) 0 (0) 0 (0) 0 (0) 0 (0)
Thyroidectomy (%)	Lobectomy Total thyroidectomy	21 (19.6) 86 (80.4)	128 (43.1) 169 (56.9)	248 (89.9) 28 (10.1)
RAI-a (%)	RAI-a No RAI-a	61 (57.0) 46 (43.0)	93 (31.3) 204 (68.7)	7 (2.5) 269 (97.5)
TSH suppression (%)	Suppressed Un-suppressed unknown	62 (57.9) 39 (36.4) 6 (5.6)	61 (20.5) 224 (75.4) 12 (4.0)	6 (2.2) 270 (97.8) 0 (0)
Recurrence (%)		23 (21.5)	18 (6.1)	3 (1.1)
Cause specific death (%)		2 (1.9)	0 (0)	0 (0)

*TNM* classification is due to UICC, *N0* no lymphnode metastasis, *N1a* metastasis to central lymph node, *N1b* metastasis to lateral lymph node

**Table 2:** The relationship between the recurrence site and RAI-a

	RAI-a	Non-RAI-a
Total number of recurrences	12	6
*Recurrence site*		
Local	2	0
Thyroid	0	1
Lymph-nodes	8	4
Lung	2	1

*RAI-a* radio-active iodine ablation

**Fig. 2:**
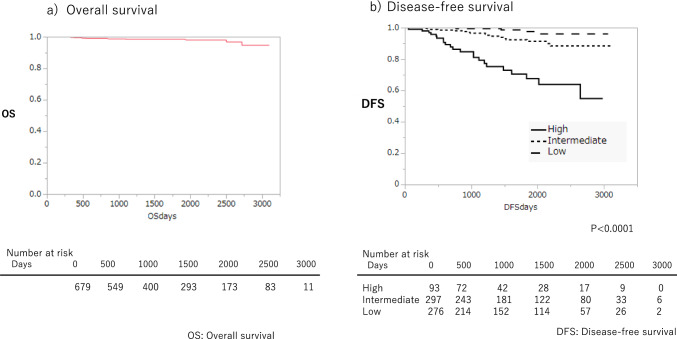
Overall survival and Disease-free survival according to risk group

Overall survival (95% Confidence interval: IC) of all the participants at 3, 5, and 8 years were 99.4% (98.8–100), 99.4% (98.8–100), and 96.3% (85.5–99.1%), respectively (Fig. 2). DFS (95% IC) at 3, 5 and 8 years were 99.4% (96.1–99.9), 97.7% (93.0–99.3) and 99.7% (93.0–99.3) in the low-risk group, 96.5% (93.0–98.2), 91.3% (85.6–94.8) and 88.3% (80.9–93.3) in the intermediate-risk group, and 81.1% (69.9–88.5), 60.6% (56.7–80.5) and 63.9% (48.5–75.9) in the high-risk group indicating statistically significant differences between all three groups (*p* < 0.0001) (Fig. 2a).

**Fig.3:**
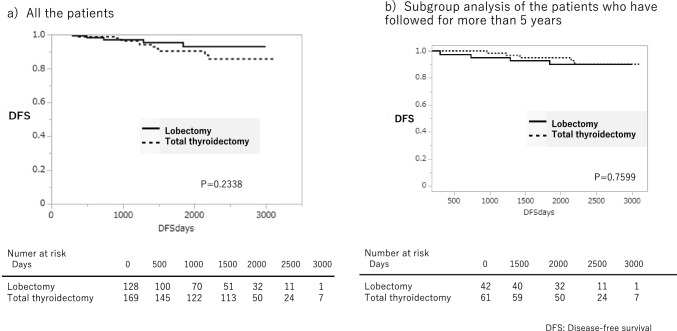
Disease-free survival following lobectomy vs. total thyroidectomy in the intermediate-risk group

**Fig.4:**
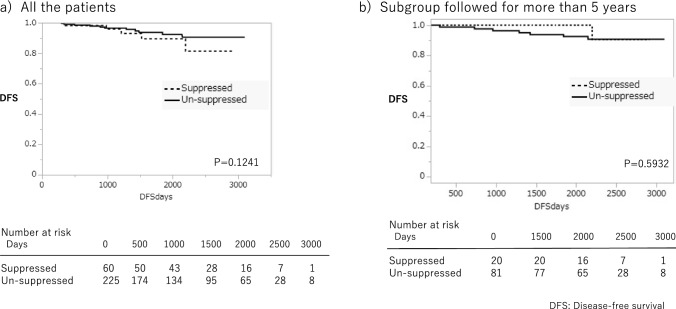
Disease-free survival with or without TSH suppression therapy in the intermediate-risk group

**Fig.5:**
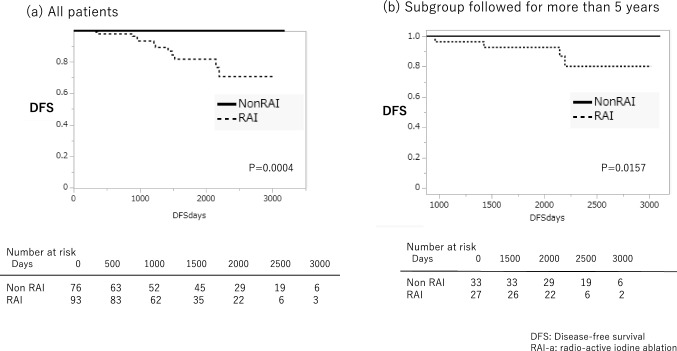
Disease-free survival with or without RAI-a after total thyroidectomy in the intermediate-risk group

In the intermediate-risk group, the calculation of DFS in each treatment category indicated no statistically significant differences between lobectomy vs. thyroidectomy (*p* = 0.2388). DFS(95% IC) at 3, 5, and 8 years were 97.2%(91.5 ~ 99.1), 95.6%(88.4 ~ 98.4), and 93.2%(83.7 ~ 97.4) in lobectomy and 95.2%(90.2 ~ 97.7), 89.7%(82.3 ~ 94.2), and 85.3%(75.4 ~ 91.7) in total thyroidectomy (Fig. 3a). Furthermore, there was no statistically significant difference in DFS in the presence or absence of TSH suppression (*p* = 0.1241). DFS(95% IC) at 3, 5, and 8 years were 93.2% (83.0 ~ 97.4), 87.8% (74.6 ~ 94.6), and 80.5%(59.4 ~ 92.1) in the TSH suppression group and 96.6%(92.7 ~ 98.%), 93.9%(88.4 ~ 96.9), and 91.0%(83.6 ~ 95.3) in without TSH suppression group (Fig. 4a). However, statistically, significant differences were seen between whether or not RAI-a was administered postoperatively. DFS (95% confidence interval) at 3, 5, and 8 years were 93.2% (84.6 ~ 97.2), 81.6% (68,3 ~ 90.2), and 70.7% (51.4 ~ 84.6) in patients with postoperative RAI-a, whereas 100%, 100%, and 100% in patients without postoperative RAI-a after total thyroidectomy, respectively. Specifically, patients who did not undergo RAI-a had a better DFS than those who underwent RAI-a postoperatively (*P* < 0.0004) (Fig. 5a). There was statistical significance in the extent of lymph node metastasis and extrathyroidal extension in all the subgroups (Tables 3, 4, 5). One hundred fourteen patients with lateral neck lymph node metastasis were in the intermediate-risk group. Of these, only 29 patients (25.4%) underwent total thyroidectomy followed by RAI-a. Seventy-six patients (66.6%) underwent total thyroidectomy without RAI-a. Only nine patients (7.9%) underwent lobectomy. Extrathyroidal extension beyond the sternothyroid muscle (Ex2) is also a risk factor for the prognosis of thyroid cancer. In the intermediate-risk group, there were 41 patients of Ex2. Thirty-two patients (78%) underwent total thyroidectomy; of these, 28 patients (88%) were administered RAI-a. One hundred sixty-nine patients underwent total thyroidectomy. Of these, 93 patients (55%) had been administered RAI ablation, and 76 (45%) had not. The relationship between the recurrence site and RAI-a showed Table 2. The most popular site was lymph nodes, but there were no differences between RAI-a and non-RAI-a (Table 5).

**Table 3:** Patient’s characteristics in Lobectomy vs. Total thyroidectomy in the Intermediate-risk group

	Number	Age mean (range)	Sex (M/F)	Tumor diameter (mm)	N (*N0/N1a/N1b*)	Extrathyroidal extension (*Ex0/Ex1/Ex2*)
Lobectomy (%)	128	46 (15 ~ 87)	32/96 (25.0/75.0)	15 (2 ~ 40)	86/16/26 (67.2/12.5/20.3)	70/49/9 (54.7/38.3/7)
Total Thyroidectomy (%)	169	52 (15 ~ 78)	44/125	10 (0 ~ 40)	66/15/88 (39.0/8.9/52.1)	77/60/32 (45.5/36.7/13.8)
*p*-value		0.0046*	0.8934	0.0343	< 0.0001*	0.0298*

*RAI-a* radio-active iodine ablation, *Ex0* no extrathyroidal extension, *Ex1* extrathyroidal extension to sternothyroid muscle, *Ex2* extrathyroidal extension beyond the sternothyroid muscle, *N0* no lymph node metastasis, *N1a* metastasis to central lymph node, *N1b* metastasis to lateral lymph node

*Statistical significance

**Table 4:** Patient’s characteristics with TSH suppression vs. Un-suppression in the Intermediate-risk group

	Number	Age median (range)	Sex (M/F)	Tumor diameter (mm)	N (*N0/N1a/N1b*)	Extrathyroidal extension (*Ex0/Ex1/Ex2*)
Suppression (%)	60	54 (21 ~ 78)	42/18 (70/30)	21.5 (4 ~ 39.7)	15/4/41 (25/6.7/68.3)	19/20/21 (31.7/33.3/35)
Un-suppression (%)	225	50 (15 ~ 87)	169/56 (75.1/24.9)	21 (1.8 ~ 40)	132/27/66 (58.7/12/29.3)	121/85/19 (53.8/37.8/8.4)
*p*-value		0.1037	0.413	0.0647	< 0.0001*	< 0.0001*

*RAI-a* radio-active iodine ablation, *Ex0* no extrathyroidal extension, *Ex1* extrathyroidal extension to sternothyroid muscle, *Ex2* extrathyroidal extension beyond the sternothyroid muscle, *N0* no lymph node metastasis, *N1a* metastasis to central lymph node, *N1b* metastasis to lateral lymph node

*Statistical significance

**Table 5:** Patient’s characteristics with RAI-a vs. Without RAI-a in the intermediate-risk patients who underwent total thyroidectomy

	Number	Age median (range)	Sex (M/F)	Tumor diameter (mm)	*N* (*N0*/*N1a*/*N1b*)	Extrathyroidal extension (*Ex0*/*Ex1*/*Ex2*)
RAI-a (%)	93	52 (21 ~ 78)	28/65 (30.1/69.9)	20 (4 ~ 39)	20/6/67 (21.5/6.5/72.0)	31/34/28 (33.3/36.6/30.1)
Without RAI-a (%)	76	52.5 (15 ~ 76)	16/60 (21.1/78.9)	16.5 (1.8 ~ 40)	46/9/21 (60.5/11.8/27.6)	46/26/4 (60.5/34.2/5.3)
*p*-value		0.9119	0.1795	0.0377*	< 0.0001*	< 0.0001*

*RAI-a* radio-active iodine ablation, *Ex0* no extrathyroidal extension, *Ex1* extrathyroidal extension to sternothyroid muscle, *Ex2* extrathyroidal extension beyond the sternothyroid muscle, *N0* no lymph node metastasis, *N1a* metastasis to central lymph node, *N1b* metastasis to lateral lymph node

*Statistical significance

We used the Cox proportional hazard model with a stepwise selection method by *P*-value to avoid the interaction between the treatment and the patient's characteristics. For the analysis, we entered variables of age, sex, tumor size, lateral lymph node metastasis, extrathyroidal extension, total thyroidectomy, TSH suppression, and postoperative RAI-a. The result showed that RAI-a is statistically significant with a hazard ratio of 5.69 (95% CI 1.998 ~ 16.21 *P* = 0.001131).

## Discussion

This study is the first retrospective report describing the results of adopting the surgical strategy recommended by the JAES 2010 guidelines. Our study showed that the JAES intermediate-risk group has a good DFS, similar to the low-risk group. On the other hand, our study could not clarify the benefit of TSH suppression therapy and RAI-a therapy after total thyroidectomy in the intermediate-risk group. DFS was lower in subjects who received RAI-a therapy than in those who did not.

Most physicians postoperatively administer TSH suppression therapy, which has been shown to prevent postoperative recurrence of well-differentiated thyroid cancer. In 2002, McGriff et al. systematically reviewed TSH suppression therapy. They concluded that it is still controversial whether or not TSH suppression therapy will prevent the recurrence of PTC. Hovens et al. undertook a prospective study of 366 well-differentiated cancer patients. They revealed that postoperative TSH levels are closely related to recurrence and survival [11]. Carhill et al. also reported that recurrence and survival risk might decrease if the postoperative TSH score is less than 3.0 [12, 13]. American Thyroid Association guidelines recommend that postoperative TSH be less than 0.5 µg/ml in the intermediate-risk group and less than 0.1 µg/ml in the high-risk group [4]. However, Wang et al. revealed no correlation between TSH suppression therapy and prevention of recurrence in patients with low- and intermediate-risk PTC classified according to ATA guidelines [14]. Sugitani et al. reported no benefit of TSH suppression therapy in preventing thyroid cancer recurrence in low-risk PTC [15].

Park et al. also evaluated the validity of TSH suppression therapy in low-risk thyroid cancer groups. They used propensity score matching to compare a TSH suppression group with a non-TSH suppression group of thyroid cancer patients who underwent lobectomy. They concluded that there was no benefit of TSH suppression therapy in low-risk patients [16]. Lee et al. also insisted that TSH suppression therapy did not affect the prognosis following lobectomy in low-risk PTC patients [17]. As for intermediate- and high-risk thyroid cancer patients, two studies examined the validity of TSH suppression therapy in 2019 [18, 19]. Both studies concluded no evidence of prolonging overall or disease-free survival by postoperative TSH-suppression therapy. In the study by Tian et al., all the participants, who were classified as intermediate- or high-risk group by American Thyroid Association (ATA) criteria, had pre-ablation serum Tg < 1 ng/ml, although the authors did not find any benefit of TSH suppression therapy on decreasing the recurrence rate. Notably, the definitions of TSH suppression were different among different studies. Park et al. defined TSH suppression as TSH levels less than 0.5 mIU/L, whereas Tian defined TSH suppression as TSH levels less than 0.1 mIU/L [16, 18].

The advantages of RAI-a in the intermediate-risk group in terms of prevention of recurrence are also unclear. An interesting finding of our study was that postoperative adjuvant RAI did not improve DFS. Still, it was associated with a relatively poor DFS. The ATA and JAES guidelines recommend postoperative RAI for high-risk patients, not low-risk ones. However, there are conflicting opinions regarding postoperative RAI for ATA-defined intermediate-risk patients, such as those with T1-3 and node-positive disease or those who are >45 years old, regarding improving DFS. As for survival, Ruel et al. reported that RAI possibly decreases 29% of the mortality risk, especially for patients >45 years old. The risk reduction of mortality is up to 36% [20]. For low-risk groups, Schlumberger et al. revealed that the completion rate of postoperative RAI was equivalent to recombinant human thyrotropin and thyroid hormone withdrawal, and 1.1 GBq and 3.7 GBq. However, they did not evaluate whether postoperative RAI would decrease the recurrence rate [21]. On the other hand, Lamartina et al. revealed in a systematic review that there was no evidence of RAI benefit in preventing recurrence for low-risk patients of differentiated thyroid cancer. There is still controversy about RAI benefits for intermediate-risk patients [22]. ATA guidelines do not recommend RAI therapy for low- and most intermediate-risk patients, but it is performed at some institutions for low-risk patients. Dhar et al. reported that even in 2020, 77% of low-risk patients received postoperative RAI-a therapy, and so did 99 ~ 100% of intermediate- and high-risk patients [23]. However, the definition of RAI-a in each study was different or unclear. The revised JAES guidelines of 2010 classified RAI-a as “Remnant ablation” (30 mCi), “Adjuvant ablation” (100 ~ 150 mCi), and “Cancer treatment” (100 ~ 200 mCi). We performed remnant ablation in our intermediate-risk patients according to these guidelines.

The appropriate surgical procedure in intermediate-risk PTC patients is also controversial. Our study did not find any benefit of total thyroidectomy in improving DFS. In the 21st century, several studies using a large-scale database have been reported to compare the overall survivals of total thyroidectomy versus lobectomy in patients with PTC, such as the Surveillance, Epidemiology and End Results (SEER) study and the National Cancer Database (NCDB),. However, no studies revealed that total thyroidectomy prolonged overall survival [24–28]. So far, three studies from Japan have compared disease-specific survival (DSS), DFS, cause-specific survival (CSS), and recurrence-free survival (RFS) of lobectomy and total thyroidectomy in low-risk groups. Neither of the studies showed any evidence of benefit for DSS, DFS, and CSS in total thyroidectomy [29–31]. Recently, Wang et al. reported the RFS of PTC patients with T2N1b cancer. Among the patients who did not receive RAI-a, recurrence-free survivals of total thyroidectomy and lobectomy were 97.7% and 97.4%, respectively, with no statistically significant difference [32]. Rajjoub et al. compared OS following total thyroidectomy to lobectomy in PTC patients with T1 ~ T4 disease. They concluded that total thyroidectomy led to a better OS in patients with more than 2 cm in diameter tumors. However, the two procedures had no statistically significant difference in patients with a tumor less than 2 cm in diameter [33].

In our study, the DFS of the RAI-a group was inferior to that of without RAI-a group, and multivariable analysis revealed that RAI-a has a risk with a hazard ratio of 5.69. Even though there was a statistically significant, we should be aware of some confounders which may affect the DFS. The PTC patients with lateral lymph node metastasis or extrathyroidal extension tend to be received total thyroidectomy, RAI ablation, or TSH suppression after the operation. More than 60% were lateral lymph node metastasis detected by US examination. In addition, the behavior of the recurrence of our study was interesting. The most major recurrence was the lymph node which can be detected by RAI and confirmed by US examination. How often and what kind of examination should be conducted as a follow-up study depend on the doctor in charge at an outpatient clinic. The patients who conducted RAI-a with more aggressive cancer might have received the structural test more frequently than the lower-risk patients. The follow-up methods' differences are supposed to confound the results.

There are some limitations to our study. First, the definition of risk groups differs between ATA and JAES guidelines. The JAES guidelines define the low-risk group as clinical T1 N0 and M0. Even though patients with less than five cervical lymph node metastases are categorized as the low-risk group according to ATA guidelines, some of the JAES-intermediate-risk groups would be classified as low-risk groups according to ATA guidelines. Therefore, our results should be carefully interpreted in Western countries. Second, the follow-up period was short. Third, this study lacked precise information on surgical pathologies such as vascular and lymphatic invasion. Since JAES guidelines did not require pathological details such as the number of lymph node metastasis, vascular and lymphatic invasion, and molecular information for the classification, some JAES-intermediate-risk groups would be classified as high-risk groups according to ATA guidelines. Forth, the strategy for RAI-a in Japan might differ from that in Western countries. In Japan, few facilities are equipped to administer RAI-a therapy (more than 100 mCi). Thus, the intermediate-risk group patients in our study can be considered to have only received remnant RAI ablation (30 mCi). Fifth, we evaluated only DFS, not OS, among the intermediate-risk group.

Thus, the intermediate-risk group of JAES includes such a heterogeneous group of PTCs that our study highlighted the differences between the JAES guidelines and the ATA guidelines.

## Conclusion

Our study did not show the efficacy of RAI-a in patients with intermediate-risk PTC. It might be possible to skip 30 mCi RAI-a for particular patients in intermediate-risk PTC, such as those negative for lymph node metastasis or minimal extrathyroidal extension. However, the surgical strategy and the follow-up method in the intermediate-risk PTC group are controversial and depend heavily on the physicians’ choice. This study implies that judging the efficacy of adjuvant therapy such as RAI or TSH suppression in intermediate-risk patients is difficult.
